# Supplementary material for: Adding the temporal domain to PET radiomic features
Source: PLoS One. 2020 Sep 23;15(9):e0239438. doi: 10.1371/journal.pone.0239438 (PMC7510999; doi:10.1371/journal.pone.0239438)
Supplement: S1 File — (DOCX) [file pone.0239438.s001.docx]

## Supporting information 1: Adding the temporal domain to PET radiomic features

Wyanne A Noortman^1,2,*^, Dennis Vriens^1^, Cornelis H Slump^3^, Johan Bussink^4^, Tineke W H Meijer^4^, Lioe-Fee de Geus-Oei^1,2^, Floris H P van Velden^1^

1. Department of Radiology, Leiden University Medical Center, Leiden, The Netherlands
2. Biomedical Photonic Imaging Group, University of Twente, Enschede, The Netherlands
3. Robotics and Mechatronics, Technical Medical Centre, University of Twente, Enschede, The Netherlands
4. Department of Radiation Oncology, Radboud University Medical Center, Nijmegen, The Netherlands

* Corresponding author

E-mail: [w.a.noortman@lumc.nl](mailto:w.a.noortman@lumc.nl) (WN)

Image Biomarker Standardisation Initiative reporting guidelines [15].

| General | | | |
| --- | --- | --- | --- |
| Aim | Identify radiomic from the temporal main that contain different information than radiomic features from static images in patients with non-small cell lung cancer of stage IB to stage IIIA (according to the TNM 7^th^ edition). | | |
| Acquisition |  | Siemens Biograph Duo | Siemens Biograph mCT |
|  | [^18^F]FDG activity | ±3.45 MBq/kg bodyweight | ±3.45 MBq/kg bodyweight |
|  | Reconstruction | ordered-subsets expectation maximization with 4 iterations per 16 subsets | TrueX, 3 iterations per 21 subsets, point-spread function-based time of flight |
|  | Filter | 5 mm Gaussian | 3 mm Gaussian |
|  | Matrix | 256×256 | 512×512 |
|  | Resolution (mm²) | 2.65×2.65 | 1.59×1.59 |
|  | Slice thickness (mm) | 3.38 | 2.03 |
|  | Slices | 47 | 109 |
|  | Time frames (1 bed position) | 20 x 5 s  5 x 10 s  10 x 15 s  10 x 30 s  16 x 75 s  8 x 150 s  1 x 600 s (static) | |
|  | Parametric images | Parametric glucose metabolic rate (${MR}_{glc}$) images were created based on tissue- and blood time-activity concentration curves using Patlak linearization, with data acquired between 15 and 60 minutes normalised Patlak-time. The image-derived input function (IDIF) was based on a 10 mL VOI of the descending aorta on which endothelial wall and calcifications were excluded to identify only blood, drawn on the images obtained during the first 60 seconds. Glucose metabolic rate was calculated as $K_{i}=\frac{C_{plasma,glc}}{{LC}_{FDG}}$ assuming $V_{b}=0$, with a lumped constant (${LC}_{FDG}$) of 1 and $K_{i}$ the [^18^F]FDG influx constant, or the slope of the Patlak plot, and $C_{plasma,glc}$, the plasma glucose concentration. | |
| Approach | The images were analysed as a volume | | |
| Software | Interpolation: MATLAB 2017b (Mathworks, Natick, Massachusetts)  Static/parametric radiomics: PyRadiomics 2.0 [18]  Dynamic GLCM radiomics: PyRadiomics 1.3 [18] | | |
| Structure | 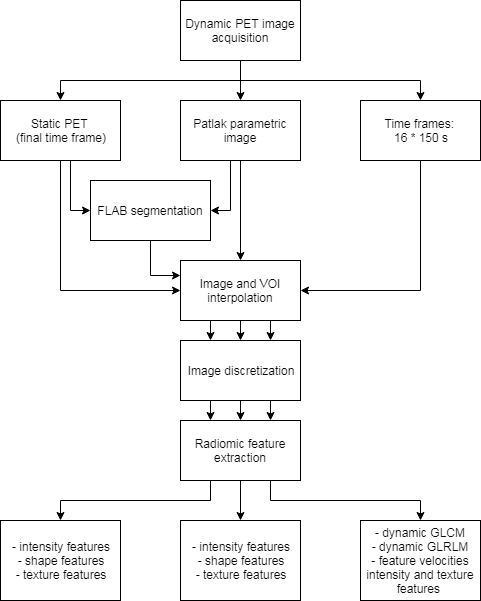  **FIGURE 1:** Pipeline for radiomic feature extraction. | | |
| Data conversion | | | |
| Procedure | Static images:  Voxel values were converted from Bq/mL to SUV, based on the injected activity with decay correction and the body weight of the patient. MATLAB 2017b (Mathworks, Natick, Massachusetts).  Dynamic images:  Dynamic images were created by combining the sixteen 75 s frames to eight 150 s frames and combining them with the eight 150 s frames to a 4D volume (sixteen frames). Voxel values were converted from Bq/mL to SUV, based on the injected activity with decay correction and the body weight of the patient. MATLAB 2017b (Mathworks, Natick, Massachusetts). | | |
| Image post-processing | | | |
| Procedure | None | | |
| Segmentation | | | |
| Procedure | Segmentation of the static and parametric tumour volumes was done using a fuzzy locally adaptive Bayesian (FLAB) algorithm [14], excluding [^18^F]FDG-avid nontumor tissue by drawing an oversized container around the tumour and surrounding tissue. The volume of interest (VOI) drawn on the static scan was also used for all frames of the dynamic scan. | | |
| Voxel interpolation | | | |
| Isotropic voxel dimension |  | Siemens Biograph Duo | Siemens Biograph mCT |
|  | Original voxel dimensions (mm³) | 2.65x2.65x**3.38** | 1.59x1.59x2.03 |
|  | Interpolated voxel dimensions (mm³) | 3.38x3.38x3.38 | 3.38x3.38x3.38 |
| Interpolation procedure | Trilinear interpolation, grids aligned by centre. | | |
| Grey level rounding | Not applicable | | |
| Grey level cut-off | Not applicable | | |
| ROI mask interpolation procedure | Trilinear interpolation, grids aligned by centre. | | |
| ROI partial volume | Integer rounding. | | |
| Re-segmentation | | | |
| Inclusion/exclusion criteria | Not applicable. | | |
| Volume resection | | | |
| Bounding box | Not applicable. | | |
| Discretization | | | |
| Discretization algorithm | Fixed bin width calculated with the Freedman Diaconis rule:$bin size=2*IQR\cdot N^{-1/3}$ [17], where IQR is the interquartile range in the VOI and N is the number of voxels in the VOI. The values for IQR and number of voxels are the mean values of all included tumour. For the dynamic scans discretization was performed for the sixteen individual time frames. | | |
| Discretization parameters | Static PET: bin width = 0.55 g/mL  Parametric PET: bin width = 1.8E-08 mol/mL/min  Dynamic GLCM, per frame: bin width: 0.26, 0.29, 0.31, 0.34, 0.36, 0.36, 0.39, 0.40, 0.42, 0.44, 0.48, 0.49, 0.50, 0.51, 0.53, 0.54 g/mL | | |
| Feature calculation | | | |
| Feature set | Static and parametric scans:   - First Order Statistics (18 features): Energy, Total Energy, Entropy, Minimum, 10^th^ Percentile, 90^th^ Percentile, Maximum, Mean, Median, Interquartile Range, Range, Mean Absolute Deviation, Robust Mean Absolute Deviation, Root Mean Squared, Skewness, Kurtosis, Variance, Uniformity - Shape based (13 features): Volume, Surface Area, Surface Area to Volume Ratio, Sphericity, Maximum 3D Diameter, Maximum 2D Diameter Slice, Maximum 2D Diameter Column, Maximum 2D Diameter Row, Major Axis, Minor Axis, Least Axis, Elongation, Flatness - Grey Level Cooccurrence Matrix (22 features): Autocorrelation, Joint Average, Cluster Prominence, Cluster Shade, Cluster Tendency, Contrast, Correlation, Difference Average, Difference Entropy, Difference Variance, Joint Energy (= Angular Second Moment), Joint Entropy, Informational Measure of Correlation 1, Informational Measure of Correlation 2, Inverse Difference Moment, Inverse Difference Moment Normalized, Inverse Difference, Inverse Difference Normalized, Inverse Variance, Maximum Probability (= Joint Maximum), Sum Entropy, Sum of Squares (=Joint Variance) - Grey Level Run Length Matrix (16 features): Short Run Emphasis, Long Run Emphasis, Grey Level Non-Uniformity, Grey Level Non-Uniformity Normalized, Run Length Non-Uniformity, Run Length Non-Uniformity Normalized, Run Percentage, Grey Level Variance, Run Variance, Run Entropy, Low Grey Level Run Emphasis, High Grey Level Run Emphasis, Short Run Low Grey Level Emphasis, Short Run High Grey Level Emphasis, Long Run Low Grey Level Emphasis, Long Run High Grey Level Emphasis - Grey Level Size Zone Matrix (16 features): Small Area Emphasis, Large Area Emphasis, Grey Level Non-Uniformity, Grey Level Non-Uniformity Normalized, Size-Zone Non-Uniformity, Size-Zone Non-Uniformity Normalized, Zone Percentage, Grey Level Variance, Zone Variance, Zone Entropy, Low Grey Level Zone Emphasis, High Grey Level Zone Emphasis, Small Area Low Grey Level Emphasis, Small Area High Grey Level Emphasis, Large Area Low Grey Area Emphasis, Large Area High Grey Level Emphasis - Neighbouring Grey Tone Difference Matrix (5 features): Coarseness, Contrast, Busyness, Complexity, Strength - Grey Level Dependence Matrix (14 features): Small Dependence Emphasis, Large Dependence Emphasis, Grey Level Non-Uniformity, Dependence Non-Uniformity, Dependence Non-Uniformity Normalized, Grey Level Variance, Dependence Variance, Dependence Entropy, Low Grey Level Emphasis, High Grey Level Emphasis, Small Dependence Low Grey Level Emphasis, Small Dependence High Grey Level Emphasis, Large Dependence Low Grey Level Emphasis, Large Dependence High Grey Level Emphasis   Dynamic scans:   - Grey Level Cooccurrence Matrix (22 features): Autocorrelation, Joint Average, Cluster Prominence, Cluster Shade, Cluster Tendency, Contrast, Correlation, Difference Average, Difference Entropy, Difference Variance, Joint Energy (= Angular Second Moment), Joint Entropy, Informational Measure of Correlation 1, Informational Measure of Correlation 2, Inverse Difference Moment, Inverse Difference Moment Normalized, Inverse Difference, Inverse Difference Normalized, Inverse Variance, Maximum Probability (= Joint Maximum), Sum Average, Sum Entropy - Grey Level Run Length Matrix (16 features): Short Run Emphasis, Long Run Emphasis, Grey Level Non-Uniformity, Grey Level Non-Uniformity Normalized, Run Length Non-Uniformity, Run Length Non-Uniformity Normalized, Run Percentage, Grey Level Variance, Run Variance, Run Entropy, Low Grey Level Run Emphasis, High Grey Level Run Emphasis, Short Run Low Grey Level Emphasis, Short Run High Grey Level Emphasis, Long Run Low Grey Level Emphasis, Long Run High Grey Level Emphasis | | |
| Feature settings | Static and parametric:  Image normalisation and distance weighting were not applied.  GLCMs were calculated in 13 directions, made symmetrical and combined to one 3D GLCM on which the features were calculated.  Dynamic GLCM and GLRLM features:  Image normalization and distance weighting were not applied.  Features were calculated in one angle, representing the temporal direction (0;0;0;1), comparing grey levels of subsequent voxels in time. Also, GLCMs were calculated in one direction, since time is not rotationally invariant. | | |
