# Supplementary material for: Adding the temporal domain to PET radiomic features
Source: PLoS One. 2020 Sep 23;15(9):e0239438. doi: 10.1371/journal.pone.0239438 (PMC7510999; doi:10.1371/journal.pone.0239438)
Supplement: S1 Fig — (DOCX) [file pone.0239438.s003.docx]

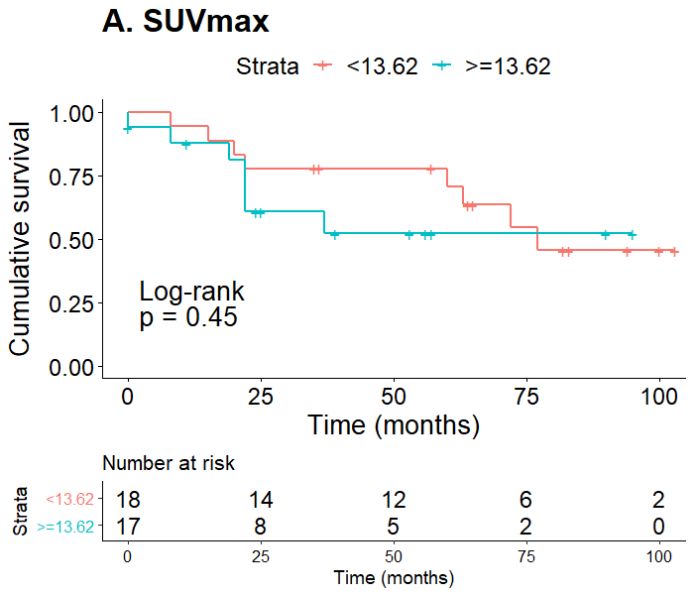

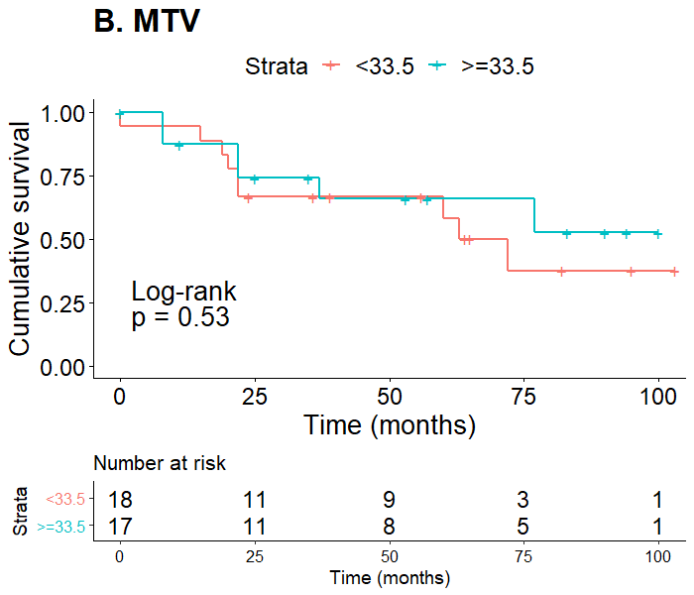

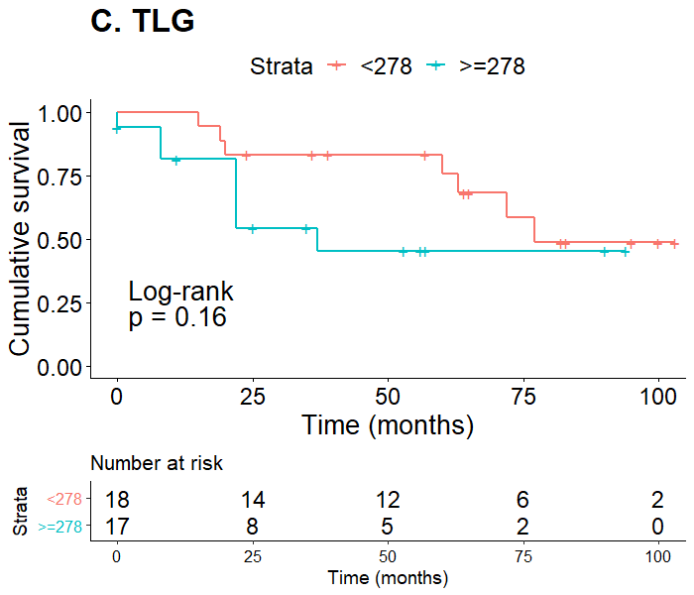

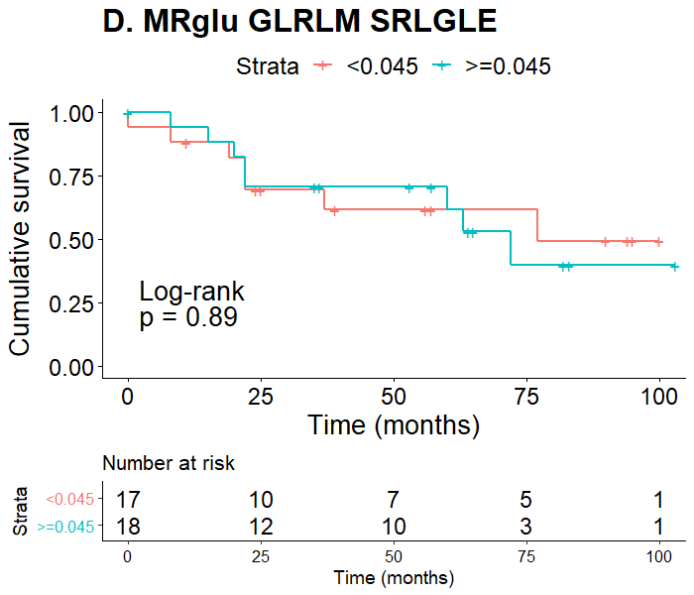

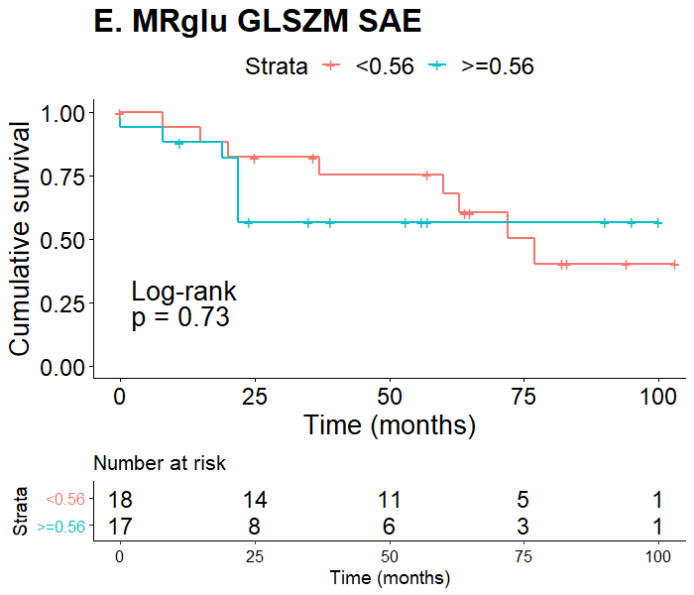

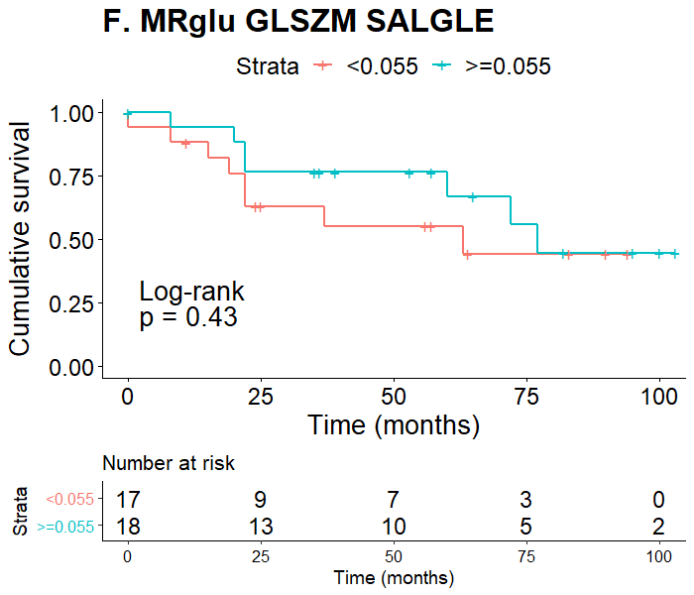

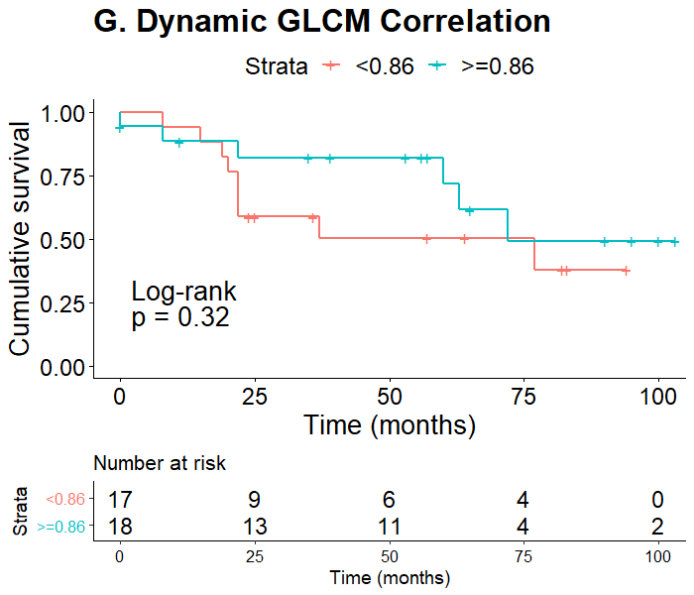

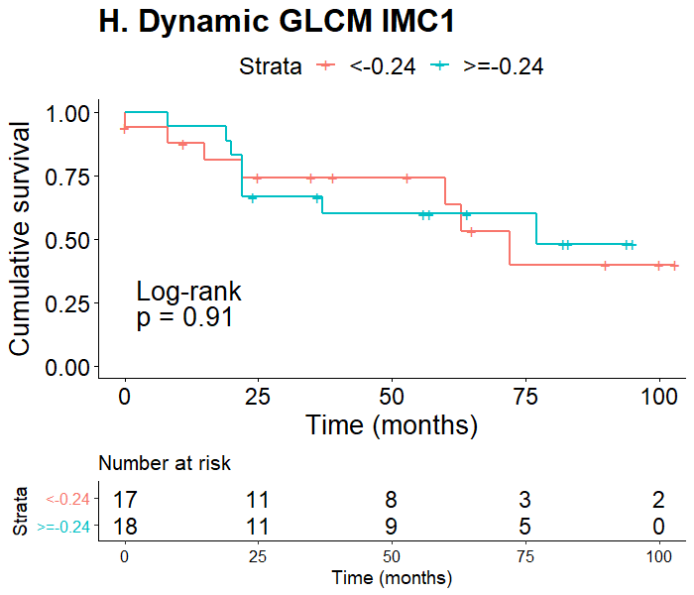

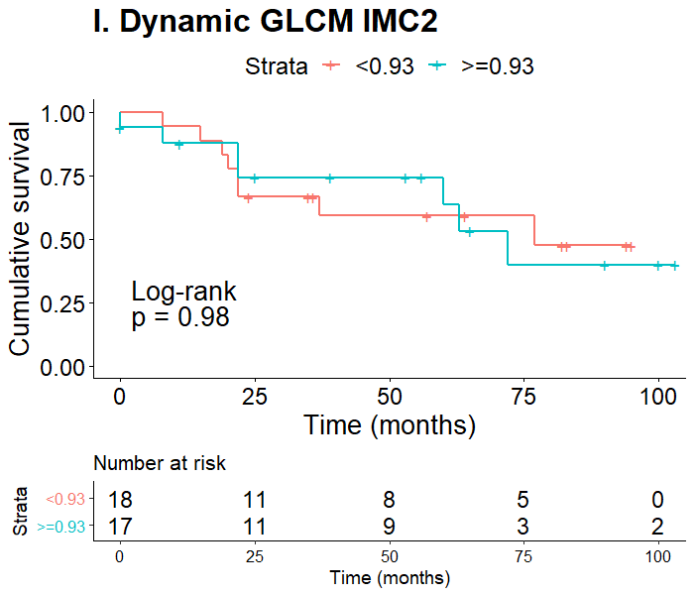

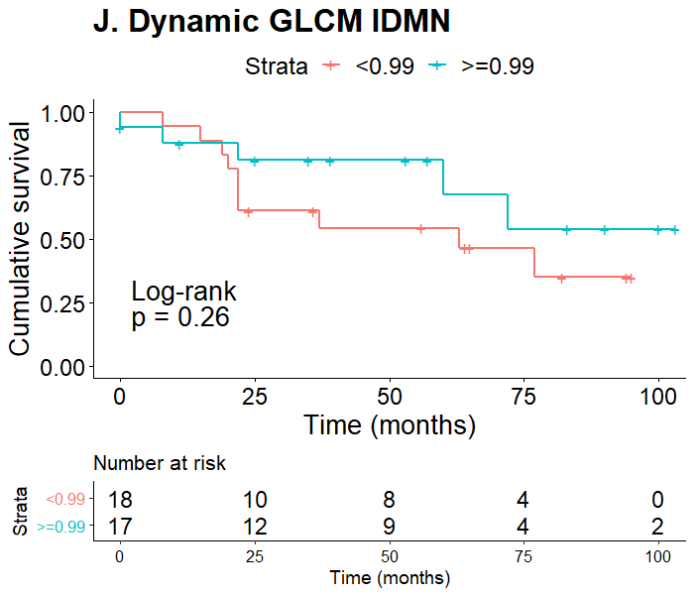

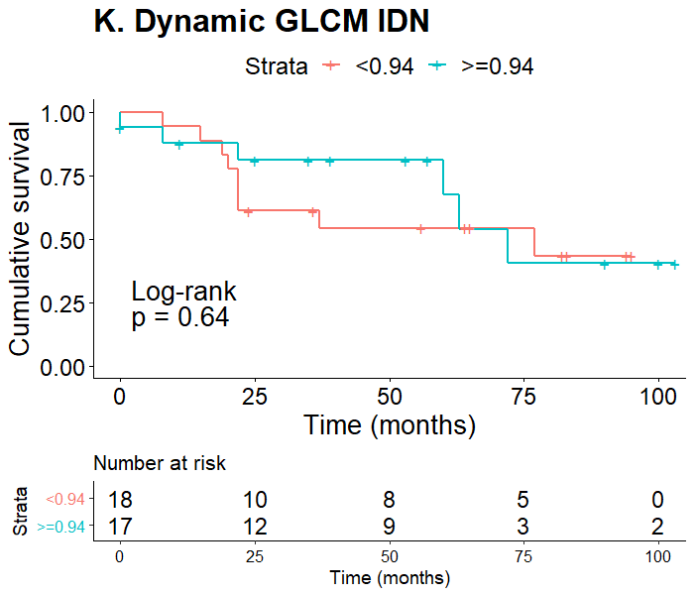


Supporting figure 1: Estimated Kaplan-Meier overall survival curves for selected features dichotomized at the median, compared using log-rank statistics. (A-C) Traditional quantitative PET features derived from static images. (D-F) Non-redundant features derived from parametric images. (G-K) Non-redundant dynamic GLCM features. SUV_max:_ maximum standardized uptake value, MTV: metabolically active tumour volume, TLG: total lesion glycolysis, GLRLM: grey level run length matrix, SRLGLE: short run low grey level emphasis, GLSZM: grey level size zone matrix, SAE: small area emphasis, SALGLE: small area low grey level emphasis, GLCM: grey level cooccurrence matrix, IMC: informational measure of correlation, IDMN: inverse difference moment normalized, IDN: inverse difference normalized.
