# Supplementary material for: Adding the temporal domain to PET radiomic features
Source: PLoS One. 2020 Sep 23;15(9):e0239438. doi: 10.1371/journal.pone.0239438 (PMC7510999; doi:10.1371/journal.pone.0239438)
Supplement: S1 Table — (DOCX) [file pone.0239438.s004.docx]

Supporting table 1: Association between histopathological characteristics and radiomic features calculated using the Mann-Whitney U test or independent samples t-test, after testing for (log-)normality. Table shows means, medians and ranges and p-values. Significant differences are indicated in bold. SUV_max:_ maximum standardized uptake value, MTV: metabolically active tumour volume, TLG: total lesion glycolysis, GLRLM: grey level run length matrix, SRLGLE: short run low grey level emphasis, GLSZM: grey level size zone matrix, SAE: small area emphasis, SALGLE: small area low grey level emphasis, GLCM: grey level cooccurrence matrix, IMC: informational measure of correlation, IDMN: inverse difference moment normalized, IDN: inverse difference normalized, AC: adenocarcinoma, SCC: squamous cell carcinoma.

|  | Histopathological subtype | | | Tumour differentiation | | | TNM stage (7^th^ edition) | | |
| --- | --- | --- | --- | --- | --- | --- | --- | --- | --- |
|  | AC | SCC | p-value | Well/moderate | Poor | p-value | Stage 1  (8 patients) | Stage 3  (7 patients) | p-value |
| Traditional quantitative PET features | | | | | | | | | |
| SUV_max_ (g/mL)  mean, median (range) | 11.4  10.8 (5.6 - 27.6) | 17.5  17.4 (9.1 - 31.0) | **0.001** | 14.2  13.3 (5.6 - 29.8) | 13.8  13.6 (8.1 - 31.0) | 0.487 | 14.2  12.2 (5.9 - 27.6) | 13.8  12.1 (8.8 - 22.2) | 0.875 |
| MTV (mL) | 52.4  35.4 (7.9 - 153.5) | 47.9  33.1 (11.5 - 180.9) | 0.777 | 49.6  36.0 (7.9 - 153.5) | 54.4  33.1 (11.5 - 180.9) | 0.891 | 28.6  22.2 (7.9 - 81.8) | 65.0  42.6 (13.5 - 180.9) | 0.167 |
| TLG (g) | 264  221 (61 - 631) | 456  297 (101 - 1669) | 0.196 | 355  269 (61 - 1233) | 433  288 (93 - 1669) | 0.613 | 202  158 (61 - 395) | 477  260 (98 - 1669) | 0.804 |
| Parametric features |  |  |  |  |  |  |  |  |  |
| GLRLM SRLGLE | 0.09  0.06 (0.01 - 0.28) | 0.04  0.04 (0.01 - 0.13) | **0.033** | 0.07  0.04 (0.01 - 0.28) | 0.05  0.05 (0.01 - 0.18) | 0.543 | 0.07  0.05 (0.01 - 0.18) | 0.09  0.07 (0.02 - 0.28) | 0.665 |
| GLSZM SAE | 0.52  0.52 (0.36 - 0.73) | 0.57  0.57 (0.40 - 0.81) | 0.155 | 0.55  0.57 (0.36 - 0.68) | 0.54  0.54 (0.34 - 0.81) | 0.857 | 0.52  0.55 (0.34 - 0.73) | 0.50  0.48 (0.36 - 0.60) | 0.721 |
| GLSZM SALGLE | 0.15  0.09 (0.01 - 0.58) | 0.05  0.05 (0.01 - 0.15) | **0.021** | 0.12  0.06 (0.01 - 0.58) | 0.07  0.06 (0.01 - 0.22) | 0.491 | 0.11  0.05 (0.01 - 0.40) | 0.09  0.08 (0.01 - 0.17) | 0.938 |
| Dynamic features |  |  |  |  |  |  |  |  |  |
| Correlation | 0.83  0.84 (0.72 - 0.92) | 0.86  0.86 (0.78 - 0.95) | 0.173 | 0.83  0.83 (0.72 - 0.95) | 0.86  0.87 (0.71 - 0.95) | 0.194 | 0.83  0.83 (0.78 - 0.91) | 0.85  0.84 (0.74 - 0.92) | 0.577 |
| IMC1 | -0.26  -0.25 (-0.37 - -0.18) | -0.25  -0.24 (-0.38 - -0.19) | 0.577 | -0.23  -0.23 (-0.33 - -0.18) | -0.26  -0.26 (-0.38 - -0.15) | 0.105 | -0.24  -0.23 (-0.29 - -0.18) | -0.25  -0.24 (-0.37 - -0.18) | 0.599 |
| IMC2 | 0.90  0.92 (0.81 - 0.97) | 0.90  0.93 (0.85 - 0.98) | 0.258 | 0.90  0.92 (0.81 - 0.98) | 0.92  0.93 (0.81 - 0.98) | 0.077 | 0.91  0.91 (0.84 - 0.96) | 0.91  0.92 (0.83 - 0.97) | 0.955 |
| IDMN | 0.99  0.99 (0.99 - 1.00) | 0.99  0.99 (0.99 - 1.00) | 0.658 | 0.99  0.99 (0.99 - 1.00) | 0.99  0.99 (0.99 - 1.00) | 0.552 | 0.99  0.99 (0.99 - 1.00) | 0.99  0.99 (0.99 - 1.00) | 0.318 |
| IDN | 0.94  0.94 (0.93 - 0.96) | 0.94  0.94 (0.92 - 0.97) | 0.934 | 0.94  0.94 (0.92 - 0.96) | 0.94  0.94 (0.93 - 0.97) | 0.354 | 0.94  (0.93 - 0.95) | 0.94  0.94 (0.93 - 0.96) | 0.300 |

Table continues on the next page.

|  | Pleural invasion | | | Overall survival | | |  |
| --- | --- | --- | --- | --- | --- | --- | --- |
|  | Yes | No | p-value | ≥65 months  (10 patients) | ≤22 months  (10 patients) | p-value |  |
| Traditional quantitative PET features | | | | | | | |
| SUV_max_ (g/mL)  mean, median (range) | 15.3  15.2 (5.6 - 27.6) | 14.7  12.6 (5.9 – 31.0) | 0.742 | 12.7  11.2 (8.1 - 29.8) | 16.9  16.0 (5.6 – 31.0) | 0.253 |  |
| MTV (mL) | 68.5  40.3 (16.5 - 180.9) | 41.8  27.5 (7.9 - 127.7) | 0.087 | 48.4  34.2 (7.9 - 180.1) | 45.5  28.1 (14.7 - 153.5) | 0.962 |  |
| TLG (g) | 529  349 (98 - 1669) | 317  202 (61 - 1233) | 0.068 | 382  214 (61 - 1233) | 323  308 (113 - 575) | 0.492 |  |
| Parametric features |  |  |  |  |  |  |  |
| GLRLM SRLGLE | 0.07  0.05 (0.01 - 0.28) | 0.06  0.04 (0.01 - 0.18) | 0.884 | 0.06  0.05 (0.01 - 0.18) | 0.07  0.04 (0.01 - 0.28) | 0.737 |  |
| GLSZM SAE | 0.55  0.55 (0.36 - 0.73) | 0.54  0.56 (0.34 - 0.81) | 0.963 | 0.50  0.51 (0.34 - 0.68) | 0.59  0.58 (0.36 - 0.81) | 0.083 |  |
| GLSZM SALGLE | 0.10  0.06 (0.01 - 0.58) | 0.09  0.06 (0.01 - 0.40) | 0.824 | 0.08  0.08 (0.01 - 0.18) | 0.11  0.05 (0.01 - 0.58) | 0.638 |  |
| Dynamic features |  |  |  |  |  |  |  |
| Correlation | 0.84  0.85 (0.71 - 0.95) | 0.85  0.86 (0.78 - 0.95) | 0.627 | 0.85  0.86 (0.71 - 0.95) | 0.83  0.84 (0.72 - 0.91) | 0.398 |  |
| IMC1 | -0.24  -0.23 (-0.38 - -0.15) | -0.25  -0.25 (-0.33 - -0.19) | 0.484 | -0.27  -0.26 (-0.37- -0.15) | -0.23  -0.23 (-0.29- -0.18) | 0.159 |  |
| IMC2 | 0.91  0.92 (0.81 - 0.98) | 0.92  0.93 (0.94 - 0.98) | 0.752 | 0.92  0.93 (0.81 - 0.98) | 0.91  0.92 (0.81 - 0.96) | 0.739 |  |
| IDMN | 0.99  0.99 (0.99 - 1.00) | 0.99  0.99 (0.99 - 1.00) | 0.866 | 0.99  0.99 (0.99 - 1.00) | 0.99  0.99 (0.99 - 1.00) | 0.510 |  |
| IDN | 0.94  0.94 (0.93 - 0.97) | 0.94  0.94 (0.92 - 0.96) | 0.862 | 0.94  0.94 (0.93 - 0.96) | 0.94  0.94 (0.93 - 0.95) | 0.493 |  |
